# Small molecule inhibitors of RAS proteins with oncogenic mutations

**DOI:** 10.1007/s10555-020-09911-9

**Published:** 2020-08-07

**Authors:** Zoltán Orgován, György M. Keserű

**Affiliations:** Medicinal Chemistry Research Group, Research Centre for Natural Sciences, 2 Magyar tudósok körútja, Budapest, H-1117 Hungary

**Keywords:** RAS proteins, GTPases, Oncogenic mutations, Small molecular inhibitors

## Abstract

RAS proteins control a number of essential cellular processes as molecular switches in the human body. Presumably due to their important signalling role, RAS proteins are among the most frequently mutated oncogenes in human cancers. Hence, numerous efforts were done to develop appropriate therapies for RAS-mutant cancers in the last three decades. This review aimed to collect all of the reported small molecules that affect RAS signalling. These molecules can be divided in four main branches. First, we address approaches blocking RAS membrane association. Second, we focus on the stabilization efforts of non-productive RAS complexes. Third, we examine the approach to block RAS downstream signalling through disturbance of RAS-effector complex formation. Finally, we discuss direct inhibition; particularly the most recently reported covalent inhibitors, which are already advanced to human clinical trials.

## Introduction

RAS proteins belong to the family of small GTPases and play a crucial role in number of important oncogenic signalling pathways. As molecular switches, these proteins are essential in regulation of cell proliferation, differentiation and survival [1, 2]. The four different RAS proteins (HRAS, NRAS, KRAS4A and KRAS4B) are encoded by three human Ras genes (HRAS, NRAS and KRAS). Presumably due to their crucial role in signalling, RAS family of genes is frequently mutated in human cancers. These mutations cause consecutive activation of signalling, hence the development of several cancers such as pancreatic, colorectal and lung malignancies. It was shown that mutations at codons 12, 13 and 61 of any RAS isoforms can be associated with cancer construction. Deep involvement of RAS proteins in cancer establishment made the discovery of RAS-targeted therapies being one of the most researched fields in the past 30 years. In this review, we are focusing on mutations occurred in RAS proteins and summarizing the small molecular inhibitors for oncogenic mutants.

## RAS proteins

The RAS superfamily contains more than 150 RAS-like genes [3], which are characterised by the presence of a catalytic G-domain [4]. Based on the sequence and functional similarities, five main branches were formed: RAS, Rho, Ran, Rab and Arf [3, 5, 6]. The RAS family can be divided into six subfamilies which are RAS, Ral, Rap, Rheb, Rad and Rit [5, 6]. The most interesting members of RAS subfamily are Harvey-Ras (H-RAS), neuroblastoma-Ras (N-RAS) and Kirsten-Ras (K-RAS). The latter has two splice variants KRAS4A and KRAS4B, which differ in their C-terminal region and therefore in the membrane localization. In human cells, KRAS4B is the dominant form; KRAS4A, however, is much more tissue restricted [7].

RAS proteins contain 188 amino acids from which the first 172-174 are nearly identical between the RAS subfamily members, with only a few differences. This is the so-called GDP/GTP-binding domain (the G-domain) that can be subdivided into two main regions: the first 86 amino acid constitute the effector lobe that is fully identical among the RAS isoforms and an allosteric lobe (amino acids 87-172) which has 86% identity. The effector lobe can be subdivided into three regions, the so-called switch I ((SW1) amino acids 30-38) and switch II ((SW2) amino acids 59-67) regions and the phosphate-binding region ((P-loop) amino acids 10-17). The last 20 amino acids (168-188) form the less identical hypervariable region (HVR) that is responsible for membrane localization and therefore for the biological activity [8].

As a plasma membrane localised molecular switch, RAS contributes in a number of signal transduction pathways through its conversion from inactive GDP-bounded to active GTP-bounded state [9]. The circulation between these two forms is helped by regulatory proteins GEFs (guanine-nucleotide exchange factors) and GAPs (GTPase-activating proteins) [10]. The formers catalyse the exchange of GDP for GTP; the latter increase the intrinsic GTP hydrolysis rate around 100,000-fold [11]. Most of the conformational changes are affecting the effector region. In the GDP-bound form, the SW1 region maintains an open conformation in order to facilitate the nucleotide exchange through guanine nucleotide exchange factors (GEFs). After depletion of the guanine nucleotide–binding pocket via GEFs, a 10-fold surplus excess of cytoplasmic GTP results nucleotide exchange [10, 12].

In response to extracellular stimuli, the active GTP-bound RAS proteins associate with numbers of effector molecules from at least 11 catalytically distinct classes [13], of which six were proved to play a role in oncogenesis, including phosphatidylinositol-4,5-biphosphate 3-kinase (PI3K), RaI-GEFs pathway and RAF serine/threonine-protein kinase [14]. Among them, PI3K and Raf are the most studied targets considering their frequent prevalence in human [15, 16].

## Oncogenic mutations of RAS

RAS proteins are the most frequently mutated proteins in cancer. Their missense mutations occur in more than 30% of human cancers [17]. The distribution among isoforms and the frequency differs across different cancer types [18]. From RAS mutations, KRAS is the most commonly mutated isoform with 86 % probability, and it occurs mostly in solid malignancies including pancreatic (more than 80%), colon (approx. 30%), lung (approx. 30%), ovarian (more than 10%) and endometrial cancer (more than 10%) [17, 19]. The most frequently mutated amino acids are 12, 13 in the P-loop and 61 in switch II, but amino acids 117, 119 and 146 in the allosteric lobe can also be affected, however, less commonly [20, 21]. NRAS mutations were observed mainly in melanoma (approx. 20%), colorectal cancer (approx. 10%) and haematopoietic malignancies (approx. 10%) most primarily at 61. HRAS mutations were reported in bladder (approx. 10%) and in cervical cancers (approx. 10%) with mutations at amino acids 12, 13 and 61 in similar extent [17, 19].

In the recent years, several studies aimed to understand the connection between these mutations and the hyperactivation of RASs. Based on the results, mutations of glycine 12 to other amino acids cause steric clashing with the so-called arginine finger of GAP, and therefore, it disrupts the GAP-mediated GTP hydrolysis [11, 22, 23]. Mutations at Gly 13 may cause similar effects, however, with lower extent because of the larger distance between Gly 13 and the arginine finger. Gln 61 is proved to help in stabilizing the catalytic water during the intrinsic hydrolization process [24]; hence, mutations at this position may cause decrease in the intrinsic hydrolysis. Moreover, it was observed that mutation on Gly 12 also results decrease in the intrinsic hydrolysis rate, which is caused by the bulky sidechain that clashes with the Gln 61. As a consequence, the active site pocket opens through further rearrangement of the surrounding switch II amino acids [25]. These mutations, however, affect negatively the GAP binding and the intrinsic hydrolysis but not influencing the GEF binding, and therefore, the activated population of KRAS is increasing upon mutations [11, 26].

## Inhibition of RAS proteins

The role of RAS proteins in numerous essential cellular processes is well known for more than three decades; therefore, a number of drug discovery programs aimed to develop inhibitors against these oncogenes. This turned to be, however, extremely challenging as the inhibition of these cancer-related hyperactivated targets would be necessary, but on the other hand, inhibition of wild-type RAS could be lethal [27]. Although inhibition of RAS proteins itself would not be crucial, which was proven by the fact that modification of Kras gene to express HRAS protein resulted viable embryos, nevertheless the substitution of inhibited KRAS with NRAS or HRAS is not possible in adults due to the tissue-specific expression of RASs [27]. Moreover, it was also reported in that wild-type KRAS has a tumour-suppressor effect in some KRAS-mutant cancers [28–31]. Hence, selective inhibition of mutant RAS proteins would be necessary.

For the interdiction of mutant RAS-induced inappropriate enzymatic activity, the formation of effective interaction through RAS and its downstream partners should be foiled. This can be performed through four main mechanisms: (i) disturbing the membrane localization, (ii) decreasing the magnitude of activated RAS proteins, (iii) disrupting the interaction with the downstream partners and (iv) stabilizing non-productive RAS protein complexes.

### Disturbing membrane association

Association of RAS to the plasma membrane is crucial for its activity and hence for the oncogenic function. Therefore, targeting this process could be an attractive solution for anti-RAS therapeutics [32, 33]. In the early stages of anti-RAS research, this direction aimed the posttranslational modifications of RAS proteins that regulate the membrane localization. One of these posttranslational modifications is prenylation that is produced by farnesyltransferases (FTases), and it is essential in oncogenic transformation [34–38]. therefore, a large number of pharmaceutical companies aimed to develop farnesyltransferase inhibitors (FTI). FTIs turned to be highly effective in numerous RAS-driven cancer cell culture and animal models; however, these results were not reflected in clinical trials. Only two small molecule FTIs were evaluated in phase III, lonafarnib [39] and tipifarnib [40] (Table 1). FTIs did not show efficacy in KRAS- or NRAS-driven cancers, while these proteins undergo alternative prenylation in FT impaired cells. This is caused by geranyl-geranyl transferase-I (GGTase-I), which is originally not the enzyme of KRAS or NRAS; nonetheless, in FT-blocked cells, covalent addition of a C20 geranygeranyl isoprenoid lipid was observed, which restored the membrane association [41, 42]. To avoid this event, another solution would be the simultaneous usage of FTIs with geranylgeranyl transferase inhibitors (GGTI) as GGTI-2417 GGTI-2418 [43] or GGTI-2147 [44] (Table 1). To confirm this idea, FTase knockout mice harbouring KRAS-G12D-driven lung cancer were treated with GGTI-2147 which efficiently reduced tumour development [45]. Although in this paradigm the impact of KRAS mutations is limited to the increased RAF and decreased p27 activity, these enzymes are responsible for hundreds of protein substrates; therefore, the off-target toxicity can be concerned [34].

**Table 1:**
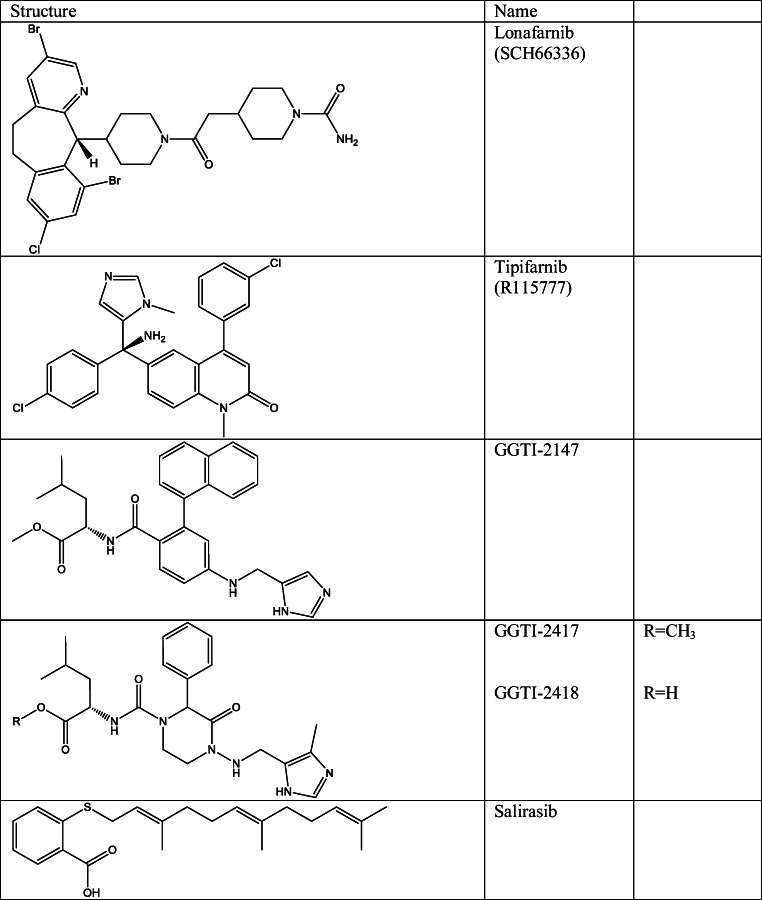

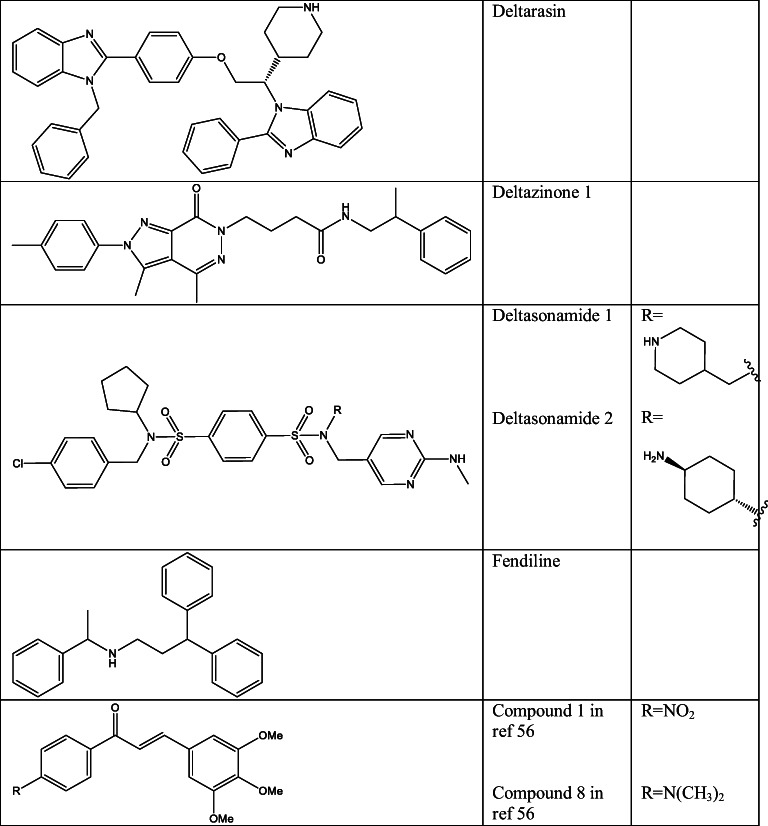
Structure of small molecule inhibitors of RAS membrane association

Further inhibitors were also developed for inhibiting RAS membrane association. One of these was salirasib (Table 1), which is a competitive inhibitor of prenylated protein methyltransferase (PPMTase) that is responsible for the methylation of the carboxyl-terminal S-prenylcysteine in prenylated RAS [46]. Additional studies found that salirasib caused reduced RAS membrane localization, stability and effector interactions [47].

Recently a more promising protein, phosphodiesterase 6 delta (PDEδ), was found to be a potential objective for disrupting RAS membrane association. This is essential for transferring the farnesylated RAS to plasma membrane and for the correct localization [43]. For the disruption of KRAS-PDEδ interaction, three different small molecules were reported. The first reported compound was deltarasin [48] (Table 1) that showed efficacy in disturbance of KRAS localization to the endomembrane in human. This compound was found with a structure-based optimization from the original hit, 1-benzyl-2phenyl-1H-benzo [d] imidazole, and showed selective binding to the prenyl-binding pocket of PDEδ with nanomolar activity (K_*D*_ = 38 nM) and inhibited oncogenic KRAS signalling *in vitro* and *in vivo* [48]. This group reported another PDEδ inhibitor deltazinone 1 (Table 1), which was highly selective against KRAS-activated pancreatic cell lines (Capan-1 (G12V), Panc-Tu-I (G12V), MIA PaCa-2 (G12C)) and less cytotoxic than deltarasin; however, this compound turned to be metabolically instable and therefore inappropriate for *in vivo* experiments [49]. A later study moreover determined that the release of KRAS forms the KRAS-PDEδ complex helped by the release factor ADP-ribosylation factor-like protein 2 (Arl2) through stabilization of PDEδ. However, Arl2 also induced the release of the high affinity PDEδ inhibitors deltarazin and deltazinone 1, which caused the necessity of micromolar concentration of these ligands to efficiently reduce cell growth. The authors also reported other PDEδ ligands deltasonamide 1 and 2 with picomolar activity [50] (Table 1). These compounds resulted in strongly reduced proliferation in oncogenic KRAS-dependent Panc-Tu-I and MIA PaCa-2 cells but less activity on KRAS-independent cancer cell lines (PANC-1 and BxPC-3). The high efficacy of these ligands on KRAS-dependent cells was able to prevent the release by Arl2 [50].

Another inhibitor, fendiline (Table 1), was disclosed, which decreases KRAS4B association to membrane but not affecting HRAS or NRAS membrane association. Fendiline is an L-type calcium channel blocker, but interestingly other L-type calcium channel blockers did not caused KRAS misslocalization suggesting that this inhibition is independent from calcium channel blockade. Fendiline did not obstruct the posttranslational modification but decreased the nanoclustering of KRAS at the plasma membrane causing uniform diffusion in different cell compartments [51, 52]. Fendiline turned to be a direct inhibitor of acid sphingomyelinase (ASM) required for the appropriate cholesterol and phosphatidylserine (PtdSer) content of inner plasma membrane, which is needed for correct localization of RAS proteins to the membrane. Reduction in plasma membrane PtdSer and cholesterol levels results in misslocalization of KRAS4A-G12V and KRAS4B-G12V; interestingly, however, supplementation of cholesterol to the cells restored KRAS4A plasma membrane localization but not KRAS4B membrane localization or nanoclustering [51], which provide evidence for at least two different operations of PtdSer on the plasma membrane [51, 53, 54]. Interestingly, fendiline was also able to inhibit signalling of H-RAS-G12V, despite clustering and PM binding are minimally affected, which can be attributed also to the cholesterol depletion of the PM [51].

More recently, another type of RAS membrane association inhibitors (as Compound 1 and 8 in ref [56] (Table 1)) were reported. These molecules are able to stimulate protein kinase C δ (PKCδ) which phosphorylate KRAS at Ser 181 causing the redistribution of KRAS from the plasma membrane (PM) to other cellular membranes [55, 56]. These molecules were capable to selectively dissociate KRAS-G12V from the plasma membrane and inhibit the growth of KRAS-mediated cancers.

### Stabilizing non-productive complexes

One interesting approach to prevent increased activity of mutant RAS proteins caused unregulated cell proliferation is stabilizing protein-protein complexes that are able to preclude the downstream signalling. Burns and co-workers reported a series of compounds (Compound 4 in ref [57] (Table 2)) that bind to a unique pocket on the RAS-Son of Sevenless (SOS) complex. This pocket is hydrophobic and can be found near to the RAS-binding region of SOS and bordered with switch II region of RAS. These compounds were able to increase the SOS-catalysed nucleotide exchange *in vitro* and regulate RAS-effector binding [57]. Interestingly, based on the X-ray structure of SOS catalytic domain, only the conformation of His905 was changed upon ligand binding [58, 59], but it could be possible that these compounds caused change in the conformational dynamics of the RAS-binding interface [1].

**Table 2:**
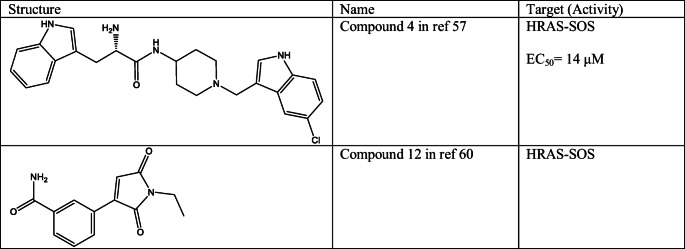
Small molecules that are stabilizing non-productive complexes of RAS

One year later, Winter and co-workers described three sets of molecules identified by crystallography-based fragment screening, which were found to bind in different regions at the RAS-SOS complex [60]. The first set of compounds was found to bind in the previously reported binding site; the second set was bound to the RAS-SOS interface. These molecules were not potent enough to detect biological activity; however, they could be starting points for further developments. Compounds in the third set were bind to the RAS protein covalently (Compound 12 in ref 60 (Table 2)) at Cys118. These latter compounds were efficient inhibitors of RAS signalling, however, only at labelling RAS-SOS complex but not RAS alone as it was shown in case of KRAS-WT, KRAS-G12C and KRAS-G12V complexes. This can be explained by the fact that the L8 loop of RAS, which contains Cys118, rotates by 180° upon SOS binding, allowing ligand binding.

### Blocking RAS-effector binding

RAS proteins are activating a number of downstream signalling pathways including PI3K-AKT-mTOR (phosphoinositide 3-kinase-AKT-mechanistic target of rapamycin) [61–64], RAF-MEK-ERK (RAF-MAPK/ERK kinase-extracellular signal-regulated kinase) [65–70] and RALGDS-RAL (RAL guanine nucleotide-dissociation stimulator-RAL) [71–74] pathways. Therefore, blocking of effector binding can be a viable solution against mutant RAS caused unregulated cell differentiation. This can be achieved in three different ways including (i) direct inhibition of RAS-effector protein-protein interactions, (ii) targeting effector protein to change its conformation in a way that it cannot bind to RAS, and (iii) changing RAS conformation prevent its binding to effector proteins. Direct inhibition of protein-protein interactions can be exceedingly difficult because of the large binding surface and the usually high efficacy of protein-protein interaction [75]; presumably, this is why no such small molecule exists for RAS-effector complexes.

For interfering of RAS-effector interaction through binding of a small molecule to effector protein, rigosertib was reported (Table 3) which binds to the RAS-binding domain (RBD) of RAS effectors preventing their interaction with activated RAS in cancer cells; regardless, it is activated by mutations of RAS proteins, or it is induced by epidermal growth factor (EGF) [76]. Since rigosertib interacts with the effector protein and therefore blocks KRAS binding, KRAS mutations have virtually no impact on its effect. In fact, the compound inhibited the binding of both wild type and mutant (G12D, G12S, G13D) KRAS proteins. The efficacy of this compound nevertheless is ambiguous, as much smaller concentrations were needed for *in vitro* binding than for *in vivo* effect. In any case, rigosertib is currently in phase III clinical trials in treatment of myelodysplastic syndromes (MDS).

**Table 3:**
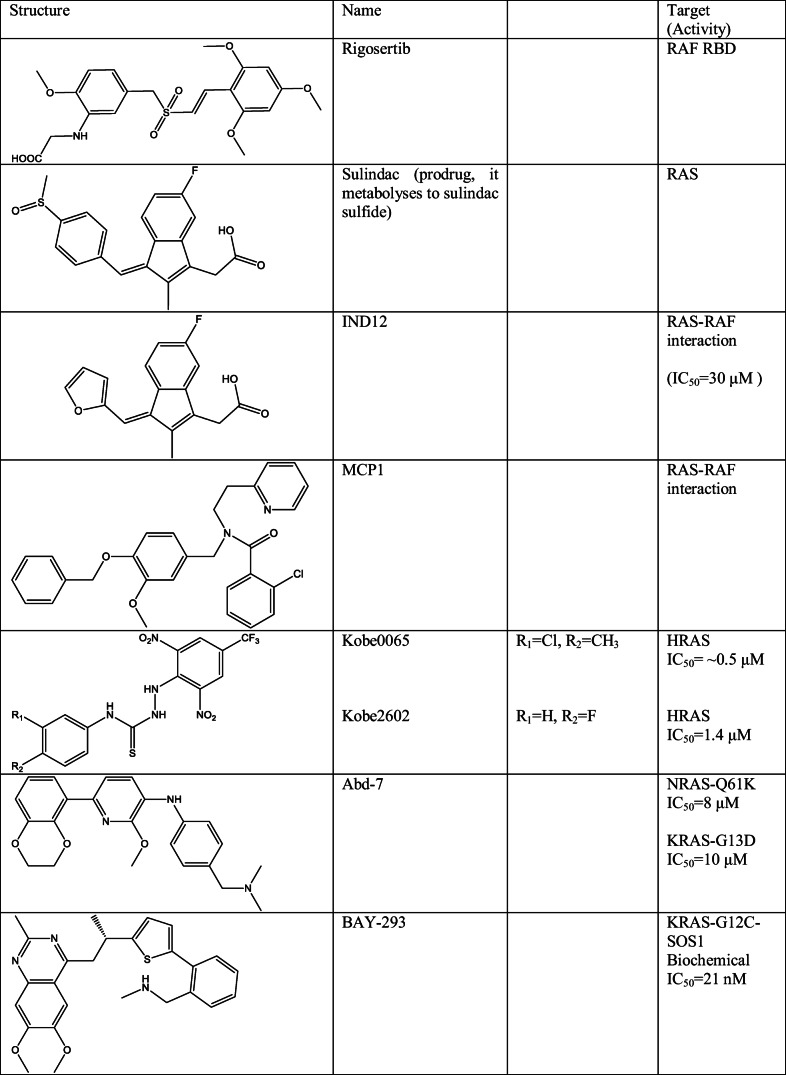
Structure of small molecules which are able to influence RAS-effector binding

A non-steroid anti-inflammatory drug (NSAID), sulindac (Table 3), was reported to inhibit RAS-induced cancer transformation and RAS/RAF-dependent transactivation through RAS binding [77]. Later, several other sulindac derivatives were reported based on phenotype-based screening (as IND12 (Table 3)). The activity of these compounds were, however, arguable because of the poorly correlating biochemical and cellular potencies [78, 79]. Moreover the direct binding of these ligands to RAS protein was also not adequately demonstrated. Furthermore, as a later study described, the inhibition of this RAS pathway activity derived from mitogen-activated protein kinase phosphatase-1 (MKP-1) and MKP-3 overexpression. These kinases are responsible for dephosphorylation of threonine and tyrosine residues in the activation loop of ERK and hence inactivation of ERK activity. Upregulated expression of MPK-1 and MPK-3 therefore may induce inactivation of the ERK activity and cause the anti-tumour activity. This mechanism may be, however, general in case of NASIDs [1, 80].

Other set of compounds (as MCP1 (Table 3)) was reported by Kato-Stankiewicz and co-workers, which was identified to inhibit RAS-RAF interaction in HT1080 (NRAS-Q61K), in PANC-1 (KRAS-G12D) and in A549 (G12S) cells in 10–20 μM range; however, these compounds also influenced ERK phosphorylation [81]. The authors also could not clearly prove direct RAS or RAF binding as in the case of sulindac derivatives.

An *in silico* screening strategy was used to identify two new RAS-binding compounds Kobe0065 and Kobe2602 (Table 3) which were able to inhibit RAS-SOS and RAS-CRAF binding *in vitro*. These compounds were promising also in cellular-based assays; hindrance of downstream phosphor-signaling of RAS and inhibition of NIH 3T3 cell transformation by HRAS-G12V were demonstrated; moreover these compounds were able to decrease tumour growth in xenograft of KRAS-G12V-mutated, human colon carcinoma SW480 cells [82].

Antibody-based target validation was disclosed by Quevedo and co-workers [83]. In this study, antibody fragments were used to inhibit RAS-effector binding, and based on antibody-binding site overlap, new small molecular inhibitors were designed that were subjected to structure-based optimization. This strategy resulted Abd-7 (Table 3) as a potential inhibitor of RAS-effector interaction. Abd-7 showed efficacy in DLD-1 (KRAS-G13D) and in HT1080 (NRAS-Q61K) cells.

More recently high-throughput and fragment screening was used in identifying nanomolar SOS1 inhibitors including BAY-293 (Table 3), which were able to block KRAS-SOS1 interaction also in case of WT and G12C mutant KRAS and hence inhibit RAS-mediated signalling pathways as RAF-MEK-ERK *in vitro* [84].

### Stabilization of inactive conformations/direct inhibition of RAS proteins

In order to stabilize RAS protein in the inactive conformation, disrupting GTP binding by targeting the GDP/GTP-binding site would be a logical strategy. Nonetheless, this would be highly challenging considering the extremely high affinity of RAS to GTP coupled with milimolar concentration of GTP in cells. Therefore, modulation of RAS proteins would be feasible at allosteric sites. However, when the first X-ray structure of HRAS virtually lacking any well-defined surface pocket was described, the researchers thought this protein undruggable.

Recent studies, however, identified new small molecular inhibitors that were able to bind non-covalently to RAS proteins and disrupt RAS functions [1, 85]. One of the first identified compounds was SCH-53239 (Table 4), which was originally designed to compete with GDP to the orthosteric-binding site of RAS [86]. Interestingly, however, this compound together with its water-soluble analogue (SCH-54292) was found to bind in a hydrophobic pocket near to the switch II (SWII) loop of RAS. Additional compounds were designed through molecular modelling based on this compounds (Compound 4 in ref [87], in Table 4). Two of these compounds also inhibited KRAS-G12R mediated but not KRAS-WT cell growth. Further development of these compounds, however, would be complicated as these all have hydroxilamin moiety which is metabolically instable and toxic; moreover, the activity of these compounds is quite low.

**Table 4:**
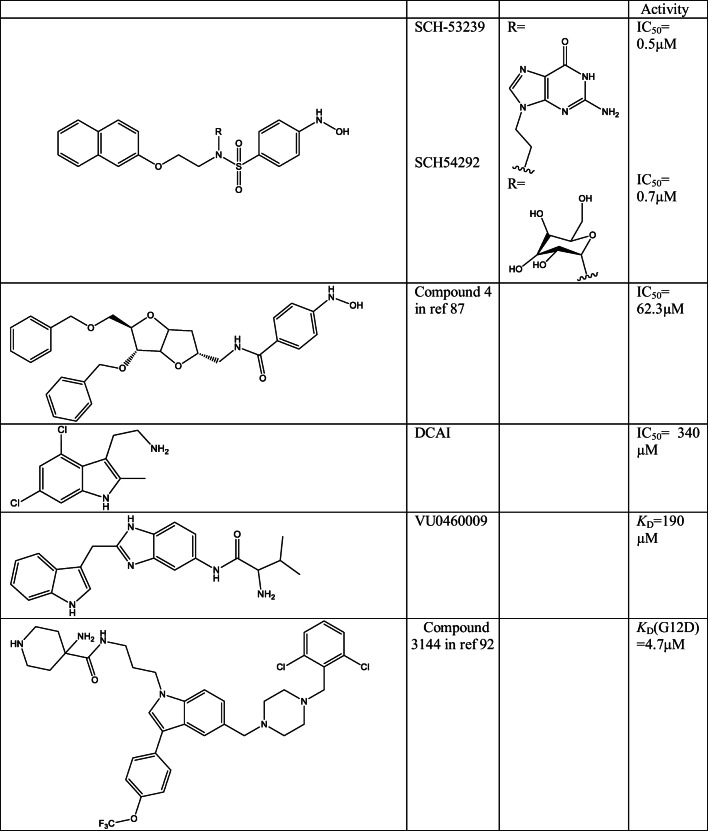
Structure of non-covalent direct inhibitors of RAS proteins

Fragment-based screening identified a compound called DCAI (Table 4), which binds in a pocket between α2 helix and β-sheet of KRAS. This compound was able to weakly block the interaction between RAS and SOS1 and inhibit RAS activation in cells [88, 89]. Another fragment-based NMR screen was performed on 11,000 compounds at the Vanderbilt University [90]. Their hits were also studied through X-ray crystallography, and it was observed that these molecules bind in the same pocket as DCAI. The previously analysed X-ray structures did not show this pocket as it is not preformed in the ligand-free KRAS. Based on the structural information, several other molecules were emerged (as VU0460009 [90] (Table 4)) with somewhat higher activity on inhibition of SOS1-mediated nucleotide exchange, through disturbance of KRAS-G12V-SOS1 complex formation. Although the authors did not test the compound on WT KRAS, the binding site is distant to the location of the investigated oncogenic mutants suggesting no selectivity over the WT protein.

*In silico* design strategy was used to develop compound 3144, a small-molecule pan-RAS ligand (Table 4). The authors find three different binding sites on the surface of KRAS-G12D and docked fragment-like and lead-like molecules to these pockets [91]. The best scored compound of the different pocket was then connected in order to increase the affinity and specificity. The best compound 3144 was tested against a number of different cell lines with WT and mutant NRAS (G13D, G13V), and selective inhibition was observed against mutant NRAS cells; moreover, it was able to decrease tumour growth in MDA-MB-231 (KRAS-G13D) and PDTALL22 (NRAS-G13V) xenograft mouse models. However, besides that, some off-target activity and toxicity were also observed.

The non-covalent inhibition of RAS proteins resulted in compounds with poor activity and in addition poor selectivity against mutant RASs. Therefore, researchers subsequently changed to mutant-specific inhibition. One strategy to achieve this is covalent modification of mutant amino acid. This can be executed the easiest way on nucleophilic cysteines. KRAS-G12C, one of the three most frequent mutations caused in cancer and as it is a non-native cysteine, therefore it can be selectively targeted. Moreover, this residue is located near to the nucleotide-binding pocket and adjacent to switch I and switch II region, which are highly dynamic regions on RAS.

The first set of covalent inhibitors that irreversibly target KRAS-G12C through covalent binding (Compound 12 in ref 92 (Si Table 1)) was described by Ostrem and co-workers [91]. They used small-molecule library screening with GDP-bounded KRAS-G12C through a tethering approach. The covalent binding was proved with X-ray crystallography, which showed that these ligands bind to a pocket between α2 and α3. This site is identical to that assumed binding SCH-54292 and was named to switch II pocket. These molecules blocked SOS1-mediated nucleotide binding and also binding of RAS to its effector proteins. Moreover, these compounds also showed selectivity in killing of G12C-mutated cells. This study showed that selective targeting of KRAS-G12C is possible, which can result high affinity ligands with low toxicity, and therefore, it opened a new field in mutant RAS inhibition.

Somewhat later another G12C inhibitor was appeared, but this compound was a GDP analogue with a so-called electrophile “warhead” (SML-8-73-1 (SI Table 1)) that was able to bind to Cys12 in mutant KRAS, but no detectable binding was observed in KRAS-WT [2, 93, 94]. Nonetheless, non-covalent binding at the nucleotide-binding site would not be feasible; this compound proved to covalently bind to KRAS-G12C even in the presence of 1 mM concentration of GDP and GTP. This compound was not able to permeate through the membrane, but its cell-permeable analogue was able to block downstream phosphorylation of ERK and AKT and had anti-proliferative effects [2].

Optimalization of Compound 12 reported in ref [95] resulted in the more broadly KRAS-G12C-specific ARS-853 (Table 5, SI Table 1). This compound is a close analogue of the earlier compound, and it is also able to bind only to the GDP-bounded KRAS. Nonetheless this ligand is faster to bind to G12C even in lower concentrations as in case of Compound 12. Binding of ARS-853 to GDP-bound KRAS-G12C prevents nucleotide changing and therefore downstream signalling through PI3K-AKT and RAF-MEK-ERK pathways. This compound can potently inhibit growth of G12C mutant cells; however, it does not inhibit non-mutated cell growth.

**Table 5:**
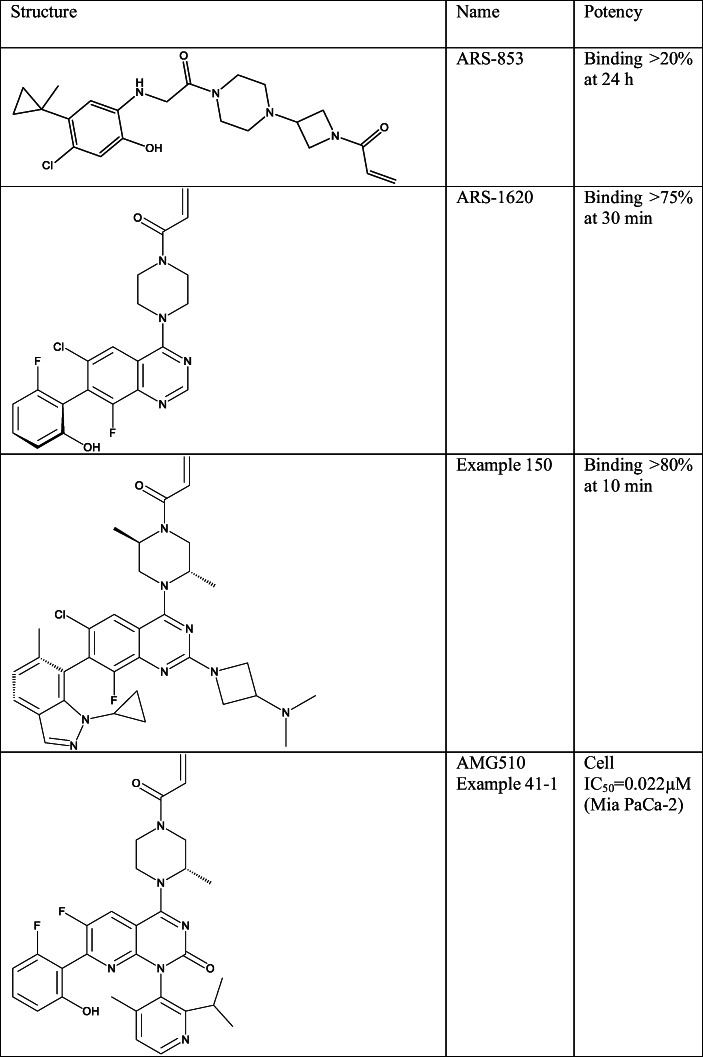

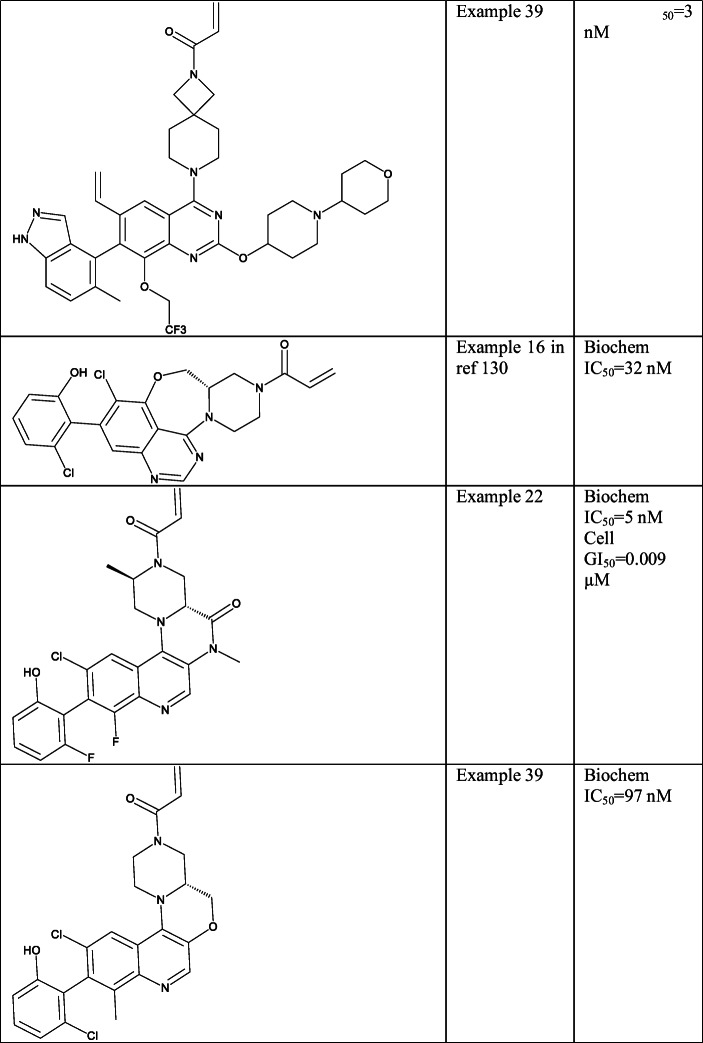

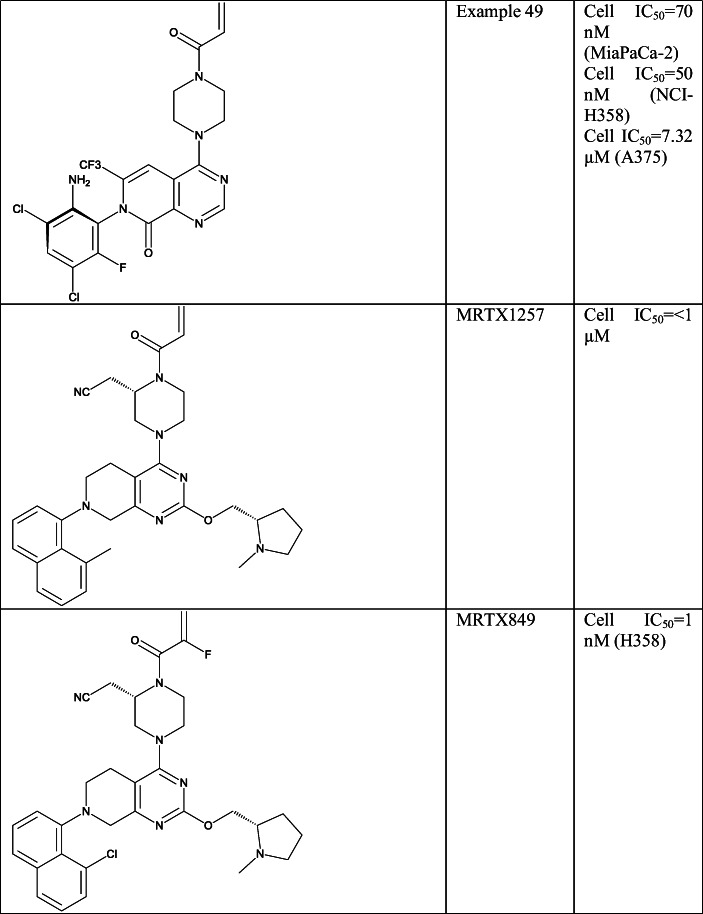

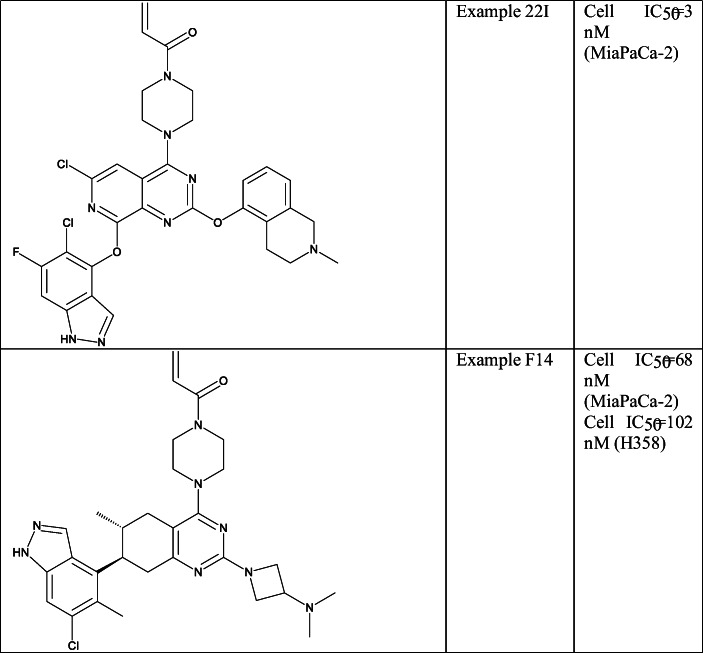
Structure of the most promising covalent inhibitors of mutant RAS proteins

The possibility of designing mutant-selective inhibitors captured the interest of numerous pharmaceutical companies, and therefore, this research area has increased significantly in the subsequent years [96]. Arexes pharma filed more than 20 patent applications from 2014 until today. In 2014, they disclosed two different patent applications, the first contained ARS-853 and its analogues with 408 examples [97]. The second was described two different scaffolds (Example I-13 and Example II-11) [98]. These scaffolds can be traced back to previous works of other researchers. Example I-13 came from Compound 4 in ref [87], but the hydroxylamine moiety of this HRAS compound was changed to the reactive warhead. Example II-11 can be originated from the scaffold of “SCH” compounds (SCH-53239 and SCH-54292).

In 2015, Araxes reported quinazoline-based inhibitors against KRAS-G12C [99] prepared from ARS-853 through scaffold hopping approach [100]. This patent is relatively large as it has 376 examples containing ARS-1620 (Table 5, SI Table 1), the first KRAS-G12C inhibitor with *in vivo* activity in xenograft models [101], and hence served as a key tool in subsequent research. Araxes itself has filed large number of patents to carefully explore this important scaffold. At first they concentrated on C2 substitution of the quinazoline ring and prepared 251 different analogues as in Example I-16 in SI Table 1 [102]. Interestingly all of these ligands have fluoro substituent on C8 position, which presumably helps to restrict rotation of the C7 biaryl bond suggesting that the activity can be connected to one of the atropisomers alone. Beside this another patent was filed in the same time also by Araxes, where C2 and C3 positions were fused with another 5- and 6-membered rings (Example 1 in SI Table 1) [103]. These compounds seemed to be less interesting based on the number of examples and the few activity data reported. Further analogues were also described in this year where biaryl groups were fused with piperazine acrylamide (Example 5 (SI Table 1) [104]). The most active compound from this set showed only moderate binding to KRAS-G12C.

Further distinct compound sets were reported in 2017 by Araxes, from which six and two were based on ARS-853 and ARS-1620 scaffolds, respectively. In the first patent, the quinazoline ring was displaced by 6,5-ring heterocycles such as imidazopyridine (Example 20 in ref [105], SI Table 1), benzofuran and benzisothiazole rings and 6,7-ring heterocycles like benzodiazepines (Example 25 in ref [105] SI Table 1). In the second patent, the position of the ring carrying the acrylamide moiety was changed to N3, and the C4 was changed to carbonyl (Example 7 in ref [106] (SI Table 1)). The other publications focused on the ARS-853 scaffold and the modification of its phenol or the glycine amide linker motif. The latter was changed to hydrazine-urea (Example 5 in ref [107] (SI Table 1)), glycine and different heterocycles including pyrazole, pyrimidine, imidazole or triazole (Example 16 in ref [108] (SI Table 1)) moieties, but there were no activity data published for these molecules. Several efforts were made to fuse the ARS-853 linker region with the piperazine moiety of the ligand into a piperidine-heterocycle (Example 1 in ref [109] (SI Table 1)). For the optimization of the phenyl moiety of ARS-853, three different approaches were reported. In the first trial, isosteric replacement was used with indazoles, indoles, benzimidazoles (Example 6 (SI Table 1)) [110] and similar heterocycles with potential H-bonding features in this region. The second application used phenol mimetic compounds based on acidic aniline groups (Example 12 (SI Table 1)) [111]. In the third patent, the phenol group was replaced by bicycles like tetrahydroquinoxalin or its mono- (Example 9 (SI Table 1)) and di-one variants [112]. Also in 2017, another huge quinazoline-based molecule set was published with 302 examples. They described further C2-substituted quinazolines, and moreover, the C4 piperazine ring was also methylated in several compounds (Example 38 and 72 (SI Table 1))[113]. The importance of this approach can be seen from the fact that all of the compounds had activity data moreover whole blood stability data was also reported for dog and monkey [113].

In the next study, the quinazoline core was further investigated. Five- or 6-membered ring heterocycles were used to replace the phenyl ring of quinazoline. Nitrogen atom was tried in all position of this ring (Example 20 in ref [114] (SI Table 1)) also as bridgehead atom [114]. Later on the quinazoline core was also changed to bicyclic groups, where 6,5-; 6,6- and 6,7-rings were presented with the second ring being non-aromatic (Example 193) [112]. For this set of compounds, azetidine acrylamide warhead was used, as in ARS-853. With this warhead in 2018, four additional sets of molecules were reported with 6,5-; 5,6-; and 6,6-membered aromatic ring systems as bezoxazoles (Example 10), indoles, benzimidazoles [115], quinazolones (e.g., Example 4 in ref [116]), benzothiazoles ( e.g. Example 4 in ref [117]), benzothiophenes [117], tetrahydroisoqinolines, and dihydroquinazolinones (Example 24) [118]. Other two types of scaffolds were disclosed where the 6,6-ring systems were connected to the azetidine acrylamide warhead or to the switch II aryl group through an amino group. In these cases, switch II aryl group was migrated from C7 to C8.

Later in 2018, the substitution of the quinazoline core was further investigated. The piperazine ring at the C4 position was substituted in 2,5 positions stereospecificly with methyl substituents (Example 1 in [119] (SI Table 1)). In addition, C2 substituents were also reconsidered; azetidines, sulphonamides and numerous different basic groups were investigated (Example 150 (Table 5, SI Table 1)) [120]. Moreover, the C7 (switch II binder) substituent was further iterated with substituted indazoles, phenols, benzoxazoles and indoles. The latest application in addition to quinazolines contains also cinnolines and quinolines, the latter with nitrile and trifluoromethyl groups at C3 or C2 positions (Example 30 (SI Table 1)) [121].

ARS-1620 has been used as a starting point by several other companies. Araxes Pharma LLC. The patents published by Amgen outlined three different core chemotypes such as 1,2-benzisothiazole (Example 11-2-2), phthalazine (Example 10-2) and quinazoline-2-one (Example 8-6-1) cores (see SI Table 1) in three patent applications. In case of the benzisothiazoles (Example 11-2-2), three main switch II binder moieties were used with 6-phenols, 5-methyl-1*H*-indazoles and 3 naphtols [122]. Moreover the possibility of changing the piperazine linker was also investigated with azetidines spirocycles, bicycles or amino-linked piperidines. Nonetheless, several substituents on the piperazine ring were also tested (methyl, fluoromethyl or difluoromethyl). The phtalazines and quinazolin-2-ones were substituted at C1 or N1 position, respectively. Here different aryls, N-linked amines, alkyls, benzyls, heteroaryls and O-alkils were used. In case of these cores, the 8 position was left unsubstituted or switched to nitrogen as in quinazolin-2-one [122].

The 8-azaquinazolin-2-one scaffold (Example 8-6-1) was further explored in the next filings. Here 2,6-disubstituted aryl rings were used at N1 at first with different groups; however, the blocked rotation of these moieties caused atropisomerism [123]. The atropisomers were separated and measured independently. This patent disclosed the structure of AMG 510 (Example 41-1(Table 5, SI Table 1)) [124], the first covalent KRAS-G12C inhibitor that reached the clinic [123, 125, 126]. In the next patent, the N1 substituent was changed to the symmetric 4,6-diisopropylpyrimidinyl group to prevent atropisomerism as in Example 8 in SI Table 1 [127]. In the patents presented by Amgen, biochemical IC_50_ and KRAS-G12C MiaPaCa-2 cellular IC_50_ values were measured for most of the examples [122, 123, 127].

The next quinazoline-based core was reported by Astellas. They used 2,7-diazaspiro[3.5]nonane linker at the C4 position and several different basic side chains at C2 position. Interestingly, the halogens at C6 were changed to cyclopropyl (Example 35 (SI Table 1)) or ethylene (Example 39 (Table 5, SI Table 1)) moiety and at C8 alkyl and aryl ethers were explored [128]. Activity data were presented for several compounds in NCI-H1373 (KRAS-G12C) xenograft mouse model.

The structures published by AstraZeneca are cyclised between the piperazine ring and the quinazoline C5 [129, 130] and quinoline C3 [131]. On the quinazoline-based analogue, 7- and 8-membered rings were investigated with an oxygen in the ring (Example 16 in ref 130 (SI Table 1)). The application of this ring was explained by the lowered binding energy of the inhibitor due to its optimal conformation [130]. On the quinoline-based analogue, 6-membered rings were considered like morpholine (Example 22 (Table 5, SI Table 1)) or piperazinone (Example 39 [132]. Several compounds from this set have favourable pharmacokinetic properties. It is worth noting that Example 39 has excellent clearance (6ml/min/kg) and bioavailability (100%) in mice.

In a later study, a pyridopyrimidinone core was published by Medshine Discovery Inc. that was also investigated by Araxes [114]. In these compounds, the N7 position was substituted with several aryl groups and C2 with numbers of basic substituents. A large number of activity data were reported for most of the compounds including inhibition in KRAS-mutated H358 (G12C), A375 (G13D), MiaPaCa-2 (G12C) cell lines, half-life with human, mice and rat microsomes and *in vivo* efficacy data. Their compound Example 49 (Table 5, SI Table 1) was compared with ARS-1620 in *in vivo* experiments, where the latter showed 52% tumour growth inhibition in 15 mg/kg, and Example 49 provided 116% in these conditions relative to the control. Moreover Example 49 showed enhanced oral bioavailability compared with ARS-1620. More recently, Araxes in collaboration with Janssen entered in a clinical trial treating tumours with G12C mutations with their orally bioavailable compound ARS-3248/JNJ-74699157 [134]; however, the structure of this compound is not disclosed yet.

Mirati Therapeutics Inc., together with Array Biopharma Inc., filed two patents with a large number of compounds carrying the tetrahydropyridopyrimidine core. In substitution, at N7 they used naphthyl, 3 or 8 substituted naphthyl groups and indazoles, at C2 basic side chains, and at C4 2,6-diazaspiro[3.3] heptanes, piperazine and 2-(cyanomethyl)piperazine. Based on X-ray structures they concluded that the basic substituents at C2 position are able to form salt bridge with Glu62, which helps to enhance the potency [135]. This was shown by testing Example 127 (in ref 136) (SI Table 1) in *in vivo* KRAS-G12C MiaPaCa-2 xenograft mouse model where it produced great efficacy; however, the oral bioavailability of this compound turned to be quite poor (2.4%). Later they demonstrated that further increase in warhead reactivity can be observed upon applying a cyanomethyl group at the piperazine ring. Based on the X-ray structure of Example 234 (SI Table 1) [136], this can be attributed to the displacement of a bound water in KRAS-G12C and an additional H-bond interaction with Gly10. Moreover, this structure also raises that no H-bonding to Asp69 is needed for the enhanced potency [137]. They also tried to revisiting the acrylamide moiety of the compounds and found 2-fluoroacrylamide as an enhanced warhead. This was also attached to their compound MRTX849 (Table 5, SI Table 1) [138–140] which is now in phase I/II clinical trial in treatment of cancers with KRAS-G12C mutation [142, 142].

Pfizer also entered into the field of KRAS-G12C inhibitors with two different sets of compounds based on the ARS-1620 structure. In these works, the pyridinopyrimidine (Example 22I (Table 5, SI Table 1)) [96, 143] core with a nitrogen at position 7 and the tetrahydroquinazoline core (Example F14 (Table 5, SI Table 1)) were applied [144]. They used basic moieties at C2 position, ether linked phenyls and heteroaryls at C8 position in the former, and indazole moieties at C7 in the latter to reach the switch II region. Cellular inhibition of KRAS-G12C in MiaPaCa-2 cell line was reported for most of the compounds in these patents.

More recently, Eli Lilly started a phase I study of LY3499446 (structure not published) as monotherapy and in combinations with other compounds including erlotinib, abemaciclib and cetuximab in solid tumours having KRAS-G12C mutations [145].

## Conclusion

RAS proteins play a crucial role in cellular processes such as regulation of cell proliferation, differentiation and survival, which was discovered about 30 years ago. The Ras gene is mutated often in cancer cases which induces unregulated cell differentiation through expression of hyperactivated RAS proteins. The previous finding that the RAS could not be targeted has been tilted, and numerous efforts have since been made to inhibit the mutated RAS proteins; however, these have not resulted in approved RAS therapies yet. Nevertheless, the new direction of direct inhibition of mutant RAS proteins with covalent inhibitors brought the new hope to the field and became one of the most investigated areas. Currently four molecules are in clinical trials including MRTX849 from Mirati, AMG 510 from Amgen, LY3499446 from Eli Lilly, and ARS-3248/JNJ-74699157 from Araxes/Janssen. Although these molecules are great promise in treatment of G12C caused cancers, the treatment of non-cystein mutated RAS proteins is still unsolved. Up to now, only a limited number of inhibitors were reported against oncogenic but other than cysteine mutant RAS protein targets. Sakamoto and co-workers reported peptides which showed 10-fold selectivity to KRAS-G12D over KRAS-WT [146]. Mirati Therapeutics started preclinical study with a “G12D inhibitor” [147] based on promising non-clinical pancreatic adenocarcinoma xenograft models. Finally, Boehringer Ingelheim entered to Phase I clinical testing of a pan-KRAS inhibitor BI 1701963 that binds to SOS, disturbs the formation of KRAS:SOS1 complex and therefore able to target a broad range of oncogenic KRAS G12 and G13 variants [148, 149]. The necessity of other RAS mutant inhibitor raises that while KRAS-G12C mutation occurs most frequently in lung cancers, in the most commonly RAS mutated pancreatic and colon cancers. KRAS-G12D and G12V mutations are significant. However, these latter may not be targeted through covalent interactions; hence, other strategies for mutant-selective inhibitors are needed. Recent advantages in *in silico* docking and fragment-based screening may also help in developing of small molecule inhibitors for these RAS mutants. These methods together with other technologies will likely yield soon small molecules that can be employed against RAS mediated human malignancies.
